# Expression characterization and transcription regulation analysis of porcine Yip1 domain family member 3 gene

**DOI:** 10.5713/ajas.19.0076

**Published:** 2019-07-02

**Authors:** Dongjiao Ni, Xiang Huang, Zhibo Wang, Lin Deng, Li Zeng, Yiwei Zhang, Dongdong Lu, Xinhua Zou

**Affiliations:** 1 Key Laboratory of Biological Feed of Ministry of Agriculture and Rural Affairs, Boen Biotechnology Co. Ltd, Guangzhou 511400, China

**Keywords:** Pig, Site-directed Mutation, Promoter Activity, Yip1 Domain, Polyinosine-polycytidylic Acid

## Abstract

**Objective:**

The Yip1 domain family (YIPF) proteins were proposed to function in endoplasmic reticulum (ER) to Golgi transport and maintenance of the morphology of the Golgi, which were homologues of yeast Yip1p and Yif1p. YIPF3, the member 3 of YIPF family was a homolog of Yif1p. The aim of present study was to investigate the expression and regulation mechanism of porcine *YIPF3*.

**Methods:**

Quantitative realtime polymerase chain reaction (qPCR) was used to analyze porcine *YIPF3* mRNA expression pattern in different tissues and pig kidney epithelial (PK15) cells stimulated by polyinosine-polycytidylic acid (poly [I:C]). Site-directed mutations combined with dual luciferase reporter assays and electrophoretic mobility shift assay (EMSA) were employed to reveal transcription regulation mechanism of porcine *YIPF3*.

**Results:**

Results showed that the mRNA of porcine *YIPF3* (*pYIPF3*) was widely expressed with the highest levels in lymph and lung followed by spleen and liver, while weak in heart and skeletal muscle. Subcellular localization results indicated that it expressed in Golgi apparatus and plasma membranes. Upon stimulation with poly (I:C), the level of this gene was dramatically up-regulated in a time- and concentration-dependent manner. *pYIPF3* core promoter region harbored three cis-acting elements which were bound by ETS proto-oncogene 2 (ETS2), zinc finger and BTB domain containing 4 (ZBTB4), and zinc finger and BTB domain containing 14 (ZBTB14), respectively. In which, ETS2 and ZBTB4 both promoted *pYIPF3* transcription activity while ZBTB14 inhibited it, and these three transcription factors all played important regulation roles in tumorigenesis and apoptosis.

**Conclusion:**

The *pYIPF3* mRNA expression was regulated by ETS2, ZBTB4, and ZBTB14, and its higher expression in immune organs might contribute to enhancing ER to Golgi transport of proteins, thus adapting to the immune response.

## INTRODUCTION

The Yip domain family (YIPF) proteins are mammalian homologues of yeast Yip1p and Yif1p; both have five similar transmembrane segments (Yip domain). They are the two members of an integral Golgi membrane protein complex that bind to the Ypt/Rab GTPases [1]. Rab GTPases are key regulators of membrane traffic [2]. The name comes from that the Rab family are annotated as Ypt proteins in yeast cells. Yip1p, Ypt-interacting protein, was first identified as a Golgi membrane protein and was found to bind the Rab GTPases including Ypt1p and Ypt31p [3]. Another integral membrane protein, Yif1p (Yip1p-interacting factor), was later identified by its ability to interact with Yip1p in a two-hybrid mass screen [1].

There are nine YIPF members in mammalian cells. Phylogenetic analyses showed that YIPF4, YIPF5/YIP1A, YIPF6, and YIPF7/YIP1B are Yip1p homologs, while YIPF1, YIPF2, YIPF3, YIF1A, and YIF1B are Yif1p homologs [4]. Knockdown of Yip1p homologs (YIPF4, YIPF5, YIPF6) caused significant reduction in expression levels of their partner Yif1p homologs (YIPF3, YIF1A, YIPF1/YIPF2, respectively), suggesting that Yif1p homologs are unstable in the absence of Yip1p homologs [5]. The knockdown of the partner (YIPF4/YIPF3, YIPF5/YIF1A) caused fragmentation of the Golgi apparatus, suggesting their involvement in maintenance of the Golgi structure [6]. Moreover, the Yip1p·Yif1p complex was also required for establishing the fusion competence of endoplasmic reticulum-derived vesicles [7], and loss function of the complex caused a block of endoplasmic reticulum (ER) to Golgi transport. Besides above nine YIPE members, researchers have identified a subunit of the Yip1p–Yif1p complex in a thermosensitive yeast strain and named it as Yos1p. Depletion or inactivation of Yos1p could block transport between the ER and the Golgi complex [8].

Human YIPF3 (hYIPF3) protein, once named killer lineage protein (KLIP-1), was expressed in nucleated hematopoietic cells, from early embryonic hematopoietic stem cells to mature adult blood lymphoid lineages. Moreover, this gene was expressed in fetal and adult GP-A^+^ erythroblasts, the fetal liver CD34^+^ subset, fetal spleen, and adult bone marrow CD56^+^ natural killer (NK) and CD19^+^ B cells, which may contribute to improving hematopoiesis and immune functions [9]. It suggested that YIPF3 might be associated with immune response; however, it is still a piece of blank paper about YIPF3 function in pigs.

In recent years, the incidence of swine diseases has been increasing. Porcine reproductive and respiratory syndrome [10] and African swine fever [11] have caused great losses to the pig industry. Numerous studies have identified differences in immune response across a range of parameters between pigs reared under the same environmental conditions [12]. Studies targeting the genetics of disease resistance are essential for pig health improvement.

As to the commercial swine industry, application of disease resistance breeding is so important that many animal scientists focus on improving genetic resistance. With this in mind, we investigated the *pYIPF3* gene to gain insight into its potential biological roles and transcript regulation traits.

## MATERIALS AND METHODS

### Ethics statement

All the animal treatment processes and the protocols we used were approved by the Animal Care and Use Committee of Guangdong Province, China. The approval ID or permit numbers are SCXK (Guangdong) 2011-0029 and SYXK (Guangdong) 2011-0112.

### 5′-flanking region cloning

To obtain the 5′-flanking region of this gene, *pYIPF3* mRNA sequence (GenBank No. NM_001245980) was blasted in the high throughput genomic sequences database (http://blast.ncbi.nlm.nih.gov/Blast.cgi). Then a porcine genomic DNA clone (GenBank No. CU928765.2) containing the gene was isolated and approximately 2,000 bp of 5′-flanking sequences was amplified with the primers YIPF-proF/R (Table 1).

### Tissue expression patterns

Tissues were obtained from three Landrace barrows. Total RNA was extracted from the tissues using Trizol reagent (Invitrogen, Carlsbad, CA, USA). RNA quality was assessed by agarose gel electrophoresis and the ND-2000 Spectrophotometer (Thermo Scientific, Waltham, MA, USA) and was discarded if the 260/280 ratio was not between 1.8 and 2.1. The RNA was then transcribed into cDNA using M-MLV reverse transcriptase (Promega, Madison, WI, USA). Real-time polymerase chain reaction (RT-PCR) was performed using Hieff qPCR SYBR Green Master Mix (YEASEN Bio, Shanghai, China) on a LightCycler 480 System (Roche, Basel, Switzerland). Beta-actin gene (β-actin) was used for endogenous control. The PCR was then performed in triplicate and the gene expression levels were quantified relative to the expression of endogenous control. The primers used in this study were list in Table 1.

### Treatment of PK15 cells with polyinosine-polycytidylic acid

To further illustrate the *YIPF3* expression changes at transcription level after virus infection, a stimulation experiment of PK15 cells with polyinosine-polycytidylic acid (poly I:C) was performed. Poly I:C, an interferon inducer, is a synthetic mimetic of viral dsRNA and induces immune responses similar to a viral infection [13]. PK15 cells were plated in 6-well plates at an amount of 2.5×10^5^ cell/well. When cells reached about 80% confluence, the medium was replaced with fresh challenge media, with or without poly I:C (Sigma-Aldrich, St Louis, MO, USA) at a final concentration of 2 μg/mL and 10 μg/mL, respectively. Cells were harvested at 6, and 12 h. Triplicate cell samples were collected at each time point. Individual RNA from the three samples at each time point was measured in duplicate.

### Subcellular localization of pYIPF3 protein in PK15 cells

In order to gain knowledge about the subcellular localization of porcine YIPF3 protein, coding sequence of *pYIPF3* gene was amplified using YIPF-C-F/R primers (Table 1) and subcloned into the *Eco*RI/*Xho*I site of the pEGFP-C1 vector (Clontech, Takara Bio Inc., Mountain View, CA, USA) to yield a mammalian expression plasmid pYIPF3-GFP. After sequencing verification, the plasmids were extracted using an Endo-free Plasmid Mini Kit (Omega Bio-Tek, Inc., Norcross, GA, USA). PK15 cells were seeded onto cover slips in 6-well plates. Transient transfections of *pYIPF3*-GFP and pEGFP-C1 (control) were performed with lipofectamine 2000 (Invitrogen, Carlsbad, CA, USA). Twenty-four hours after transfection, the cells were fixed at 37°C for 15 min with 3.7% formaldehyde, and then incubated with 10 μM Hoechst 33342 for 10 min at room temperature. Finally, the images were obtained and analyzed using the Olympus FluoView FV1000 Confocal Microscope (Olympus, Tokyo, Japan).

### Dual-luciferase reporter assay and site-directed mutagenesis

To determine transcription start sites (TSS) of *pYIPF3*, a primer-extension RT-PCR method was utilized [14,15], which is a procedure that employs a same antisense primer located in exon 1, and various overlapping sense primers ranging from nt −27 to nt +51 (Table 1). The PCR products were subcloned into the *Xho*I/*Hin*dIII site of pGL3-Basic (Promega, USA). The plasmids were co-transferred into 293T cells in quadruplet with an internal control pRL-TK (Promega, USA). Empty pGL3-Basic vector with pRL-TK was also transfected in parallel as an external control. Twenty-four hours after transfection, the activities of firefly luciferase in pGL3 and Renilla luciferase in pRL-TK were measured on BioTek Synergy HT Multi-Mode Microplate Read (BioTek, Winooski, VT, USA).

According to the result, fragments with highest activity were selected to be analyzed using the PROMO web tool and TFSEARCH. Transcription factor binding sites with high scores and interesting characteristics were mutated using QuickChange Lightning Site-Directed Mutagenesis Kit (Stratagene, LaJolla, CA, USA) with three pairs of primers (Table 1), multisite-directed mutagenesis was also performed using multisite-directed mutagenesis kit (Stratagene, USA) with above primers. After sequencing verification, firefly luciferase and Renilla luciferase activities of these mutational vectors were measured as above.

### Electrophoretic mobility shift assay

Nuclear protein extracts were prepared from 293T cells using Nuclear and Cytoplasmic Extraction Reagents (Thermo Scientific, USA). The protein concentration of each nuclear extract was detected by Pierce BCA Protein Assay Kit (Thermo Scientific, USA). All probes with 22 nucleotides were labeled at 5′ end with Biotin (Table 1, Probes). In the competition experiments, unlabeled oligonucleotides or mutated oligonucleotides without Biotin were added in 50-fold excess. When testing the effect of antibody on the mobility complex, 5 μL of the antibody was added to the nuclear extracts prior to the labeled probes. The primary antibodies of ETS2 (sc-351 X, Santa Cruz Biotechnology, Santa Cruz, CA, USA), ZBTB4 (sc-514883, Santa Cruz Biotechnology, USA) were added to the binding reactions to perform super gel shift experiments. LightShift Chemiluminescent EMSA Kit (Thermo Scientific, USA) was used in the electrophoretic mobility shift assay (EMSA), and the protocol was performed according to the paper [16].

## RESULTS

### Porcine YIPF3 highly expressed in lymph

Analysis of the *pYIPF3* cDNA sequence revealed that it encoded a protein of 347 residues with a highly conserved Yip1 domain in mammals. Further, there are five transmembrane domain distributed between residues Ile-149 and His-294 (data not shown) as predicted by TMHMM (http://www.cbs.dtu.dk/services/TMHMM/).

**Figure 1:**
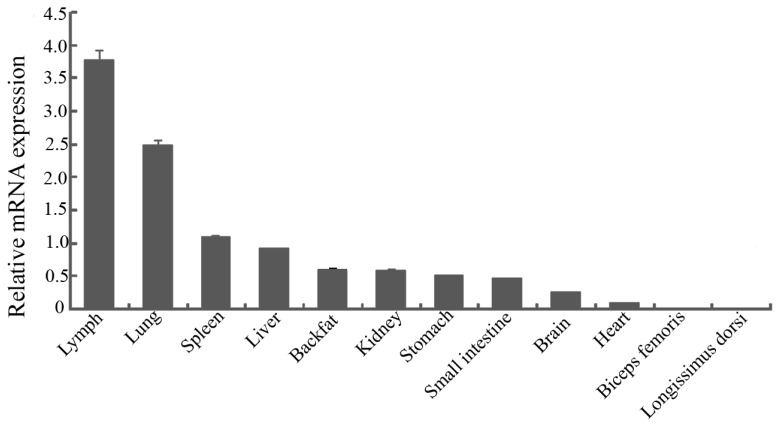
Expression analysis of porcine Yip1 domain family member 3 gene in different tissues. Error bars represent standard error of the mean value of relative expression.

The expression pattern of *pYIPF3* mRNA showed that it has highest expression level in lymph, followed by lung, spleen and liver. Lower expression levels were detected in backfat, kidney, stomach, small intestine, brain and heart, whereas almost no expression in longissimus dorsi and biceps femoris (Figure 1).

### Porcine YIPF3 mainly localized in Golgi apparatus

**Figure 2:**
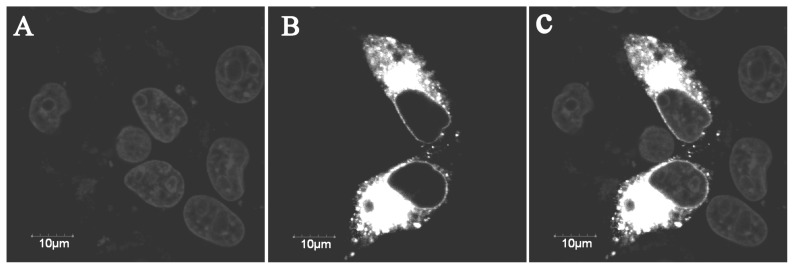
Subcellular localization of the *pYIPF3*-GFP fusion protein. Light gray refers to the nucleus (A). Bright white area is pYIPF3-GFP fusion protein (B). The overlay image was produced by merging both signals together (C). *pYIPF3*, porcine Yip1 domain family member 3; Bars represent 10 μm.

The YIPF3 fusion protein was found to distributed in Golgi apparatus and cytomembrane (Figure 2), which was consistent with its transmembrane domain predicted by TMHMM. Moreover, many documents about the location and function of yeast Yip1p and Yif1p also supported this result. Green fluorescence was detected throughout the control cells transfected with GFP vector alone (data now shown).

### Porcine YIPF3 was upregulated after stimulation with polyinosine-polycytidylic acid

**Figure 3:**
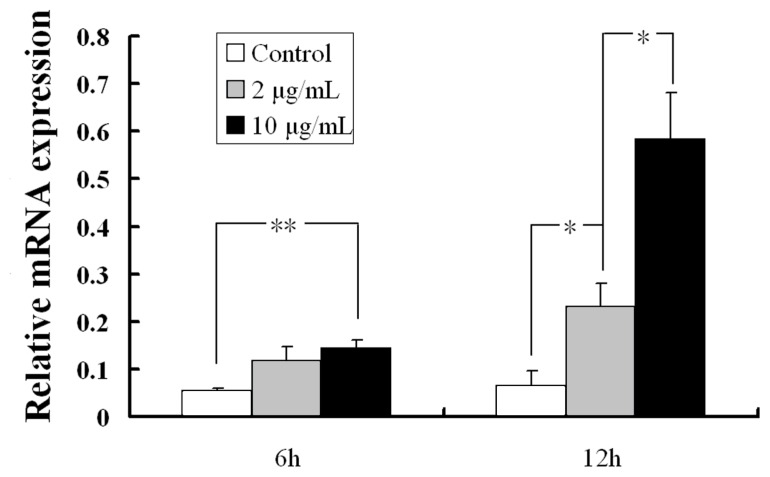
*pYIPF3* mRNA expression changes in PK15 cells after stimulation. Results are from the calculated average±standard error of three samples in same treatment. *pYIPF3*, porcine Yip1 domain family member 3. The significance of different treatments was calculated using Student’s T-test. (* represents p=0.05, ** represents p=0.01).

As described previously, *hYIPF3* gene usually expresses in fetal spleen, adult bone marrow CD56^+^ NK and CD19^+^ B cells, which drove us to further investigate its response under mimic viral infections. Our results demonstrated that *pYIPF3* mRNA in control cells was only occasionally detected. Transfection of poly (I:C) led to a significantly higher expression compared with the control. Moreover, the levels of mRNAs were markedly induced under administration of high concentration (10 μg/mL) poly I:C rather than low concentration (2 μg/mL) (Figure 3). In contrast to 6-hour poly (I:C) stimulation, the 12-hour stimulation provided higher mRNA expression levels. As a result, it seems that poly (I:C) triggered *pYIPF3* expression in a time- and concentration-dependent manner.

### Determination of transcription start sites

**Figure 4:**
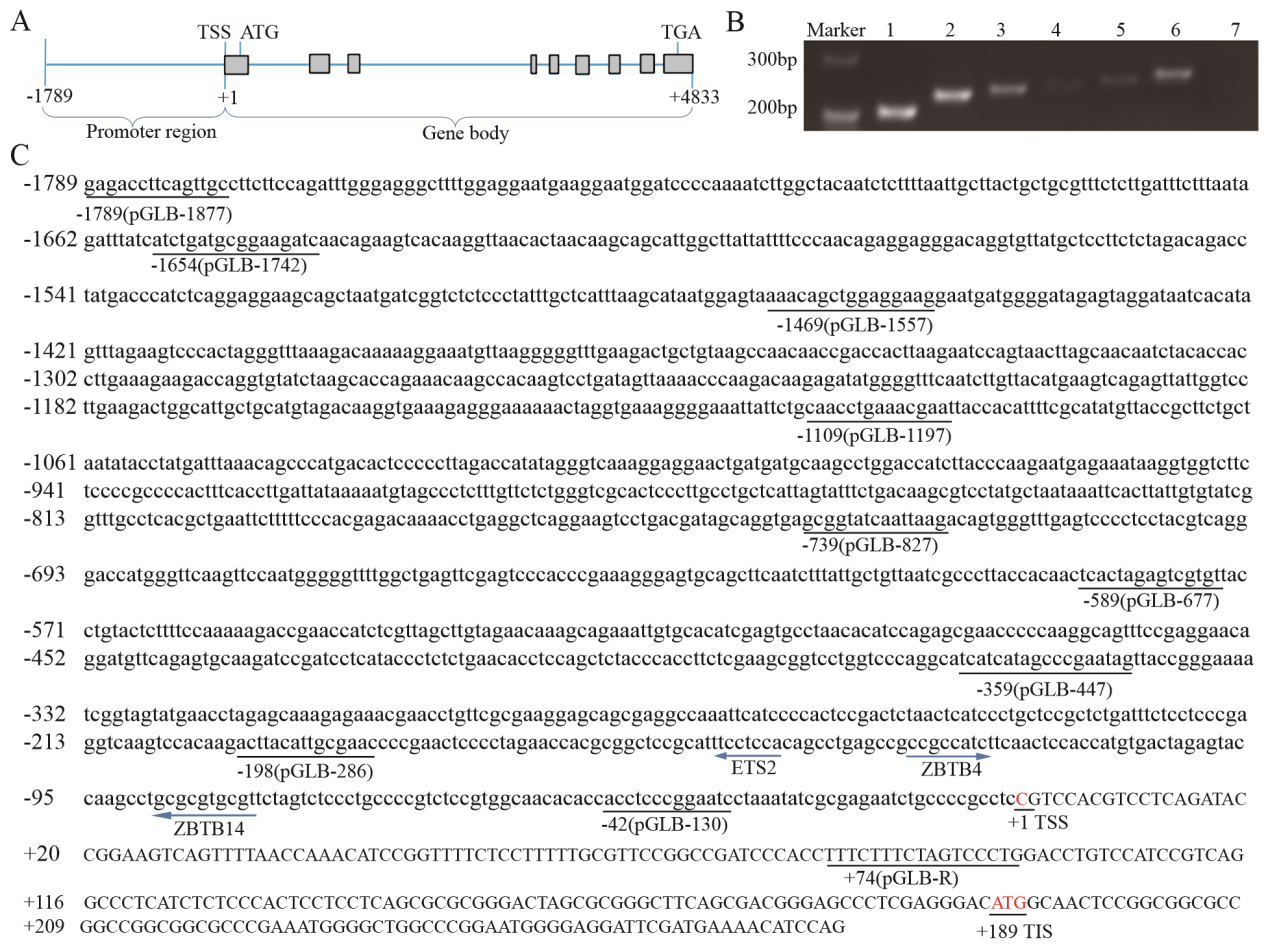
The genomic structure and promoter sequence of porcine *YIPF3* gene. (A) Schematic representation of the genomic organization of *pYIPF3* gene. The exons are colored in grey box. (B) Identification results of transcription start site using PCR. The PCR product of lane 1 is generated using the former primer YIPF-TSS(1)F and common primer YIPF-TSSR (Table 1). The Lane 2 is from YIPF-TSS(2)F, and so on. (C) Structure of the *pYIPF3* promoter region. Underlined sequences indicate primers for pGLB-1877~130 constructs. The left arrow represents the transcription factor binding to the antisense strand, while the right arrow represents binding to the sense strand. Exons are in uppercase and 5′ flank region of TSS in lowercase. *YIPF3*, Yip1 domain family member 3; PCR, polymerase chain reaction; TSS, the transcription starts site; ATG, translation initiation codon; TGA, translation stop codon; TIS, translation initiation site. The number of oligonucleotides is relative to the TSS (C is assigned number +1) of *pYIPF3*.

Like human homologous genes, *pYIPF3* has 9 exons, and the total length of the gene body is about 4,833 bp (Figure 4A). In which, the potential TSS was denoted as +1. Electrophoresis showed that all sense primers up to primer 6 yielded PCR products, indicating that these sequences were part of *pYIPF3* mRNA, whereas the 7th sense primers failed to yield PCR products indicating that it was not part of *pYIPF3* mRNA (Figure 4B). Based on the RT-PCR results, it was concluded that the TSS might distribute on regions nt −27 to nt −14, located between the 3′ ends of primers YIPF-TSS(6)F and YIPF-TSS(7)F. Agreeing with this, the “C” nucleotide of TSS (Figure 4C) predicted by the promoter predictor (http://www.fruitfly.org/seq_tools/promoter.html) was actually found in this region with highest score 0.98.

### Transcription activity analysis of pYIPF3 promoter

**Figure 5:**
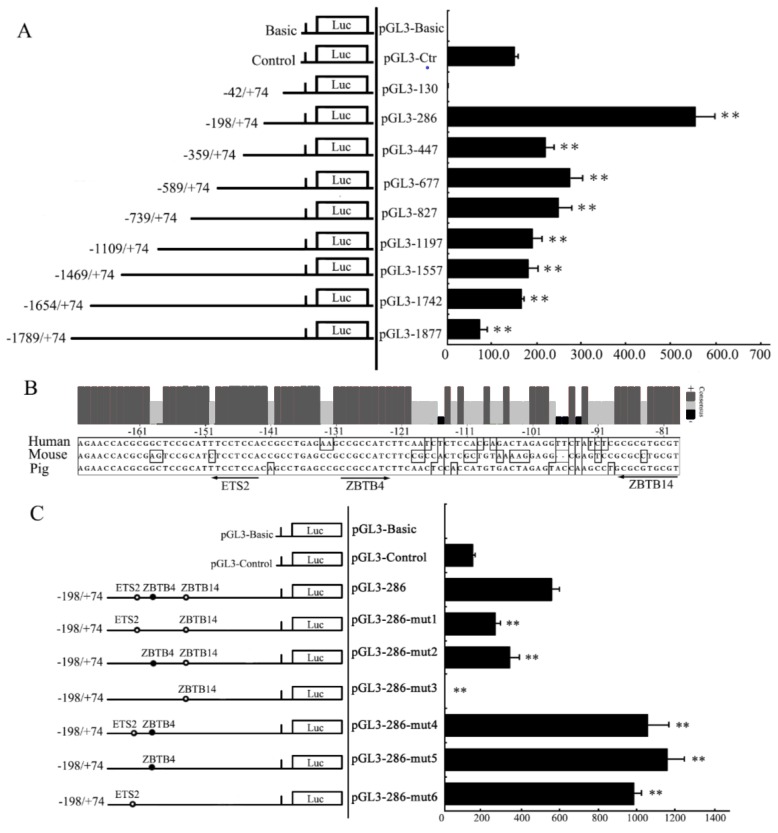
Segmental deletion analysis for *pYIPF* promoter activity. (A) The transcription activity analysis for 5′ truncated fragments of *pYIPF* promoter. The pGL3-Basic and pGL3-Control used as negative and positive control, respectively. (B) Multiple core promoter sequence alignments of porcine, human and mouse *YIPF3* genes. Different promoter nucleotides among species are presented with boxes. (C) Transcriptional activity analysis for mutational core promoter fragments of *pYIPF3*. No transcription factor labeled indicated that the site had been mutated. Hollow circle and solid circle represent the transcription factor binding to the antisense strand and sense strand, respectively. The number of each fragment is relative to the TSS (+1). These labels (130, 286, 447, etc.) represent the constructs harboring corresponding length of insert fragment. The X-axis of the graph indicates fold-activation to normalized luciferase activities. *pYIPF3*, porcine Yip1 domain family member 3; TSS, the transcription starts site. The results are represented as means±standard error of at least three independent experiments. ** represents p=0.01.

To find the important region required for transcription activity within the 5′-flanking sequence of *pYIPF3* gene, the DNA clone extending from nt −1,789 to nt +74, was truncated into serial fragments and inserted into the pGL3-Basic vector (Figure 4C). As shown in Figure 5A, all constructs pGL3-1877 to pGL3-286, but not pGL3-130, presented high transcription activity. This result suggested that pGL3-130 lacks important activation elements mediating *pYIPF3* transcription. The longer construct pGL3-286 displayed a highest transcription activity than the others, indicating that the core promoter is located from −198 to −42 (Figure 5A). Using the prediction tool of transcription factor binding site, we found that this fragment harbors three potential binding sites for multiple transcription factors including ETS2 (at −150 to −143 of antisense strand), ZBTB4 (at −130 to −123) and ZBTB14 (at −88 to −79 of antisense strand), which are all highly conserved among three species (Figure 5B). Deletion of ETS2 or ZBTB4 binding sites of pGL3-286 caused 39% or 52.5% drop of transcription activity, respectively, and almost abolishment of promoter activity was observed in the double-deletion plasmid pGL3-286-mut3 (Figure 5C). These experiments highlight the transcription enhance roles for both ETS2 and ZBTB4. On the contrary, mutation of ZBTB14 binding site resulted in prominently increase of transcription activity even when either of ETS2 and ZBTB4 binding sites was mutated simultaneously (Figure 5C).

### Transcription binding sites confirmed by electrophoretic mobility shift assay

**Figure 6:**
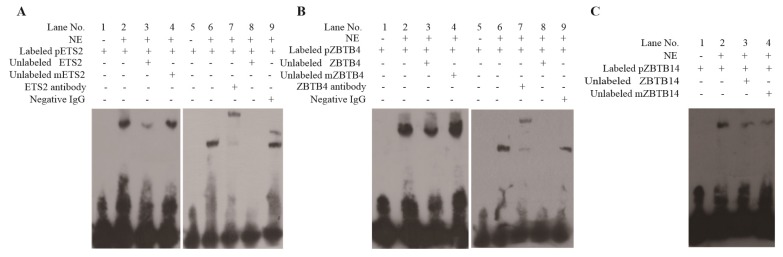
The confirmation of binding sites for three transcription factors in vitro. (A) Binding sites confirmation for ETS2. (B) Binding sites confirmation for ZBTB4. (C) Binding sites confirmation for ZBTB4. NE, nuclear extracts from 293T cells. Labeled pETS2 represents the binding sequence of ETS2 was labeled by Biotin. mETS2 means the binding sequence of ETS2 has been mutated. Similar explanation for pZBTB4 in B and for pZBTB14 in C. Competitive assays were performed using 50-fold molar excesses of unlabeled oligonucleotides. Negative IgG is used for testing nonspecific binding. ETS2, ETS proto-oncogene 2; ZBTB4, zinc finger and BTB domain containing 4; IgG, immunoglobulin G.

Our study found that the three binding sites were crucial for promoter activity of *pYIPF3*. Thus, these binding sites were used as probes in EMSA. Obviously, the probes corresponding to the three cis-elements were combined by unknown proteins, because they all had a lag in the lane 2 compared to lane 1 (Figure 6). Compared with lanes 2, the bands of all lanes 3 in Figure 4 became less intense in terms of competition with unlabeled wild-type probe. It indicated that the binding of probes for proteins was specific. Consistent with expectations, the mutation probes did not weaken the bands (Figure 6A, 6B).

As there was no suitable antibody for ZBTB14, super shift assays were only performed for ETS2 and ZBTB4 potential binding sites. The results clearly showed that the bands were significantly lagged when the antibodies were added (lane 7 in Figure 6A and 6B), which indicated that the probes can be combined by ETS2 and ZBTB4 proteins, respectively. When immunoglobulin G antibody was added, there was no similar lag band, which further indicated that the binding was specific (lane 9 in Figure 6A, B).

## DISCUSSION

Our results provided evidence that porcine YIPF3 is located in Golgi apparatus, and its mRNA showed a higher expression levels in lymph, lung, spleen and liver which are important organs related to immune system. As differential expression of genes in different tissues suggests a direct or indirect relationship between these genes’ function and these tissues [17], high expression of *pYIPF3* in these immune organs suggested that these tissues need more YIPF3 to maintain their function in normal physiological activities. Consistently, there are always a lot of immune related genes highly expressed in immune organs [18]. In addition, when PK15 cells were stimulated by poly (I:C) for different times with different final concentrations, the mRNA expression profiles of *pYIPF3* displayed in a time- and concentration- dependent manner. The expression pattern of *pYIPF3* in response to poly (I:C) was consistent with many immune-related genes such as interferon (*IFN*) [19], guanylate binding protein 1 (*GBP1*), and *GBP2* genes [20].

Transcription element analysis and site-directed mutagenesis assay showed that deletion of either ETS2 or ZBTB4 binding site would lead to decrease in transcription activity of *pYIPF3* promoter. ETS2, belonging to the ETS family which could regulate telomerase activity [21], was highly expressed in prostate cancers [22]. As an important transcription factor with mitogenic and oncogenic activity, high expression of ETS2 predicted poor prognosis in acute myeloid leukemia patients undergoing allogeneic hematopoietic stem cell transplantation [23], but also its down-regulation would inhibit the invasion and metastasis of renal cell carcinoma cells [24]. As a very important transcription activator, ETS2, which was closely related to immunity, might play an important role in the immune response that *YIPF3* participated in.

In, general, ZBTB4 usually acted as a transcription repressor, and was frequently downregulated in cancer [25]. Depletion of ZBTB4 promoted cell cycle arrest in response to activation of p53 and suppressed apoptosis through regulation of P21CIP1 [26]. Moreover, ZBTB4 restoration would suppress the tumor growth in mice [27]. But in this study, ZBTB4 showed more transcription activation for *YIPF3* expression. The specific role remains to be verified by further experiments.

ZBTB14, also known as ZF5, usually acted as a transcrip tional repressor, which inhibited DNA binding and mediated dimerization [28]. In addition, ZBTB14 was regarded as novel regulators of immunometabolic signals from skeletal muscle [29]. Here, our experiments further confirmed that ZBTB14 acted as a suppressor. Only with its repression function, can ETS2 and ZBTB4 guarantee the stable expression of *pYIPF3*.

In summary, our study showed that *pYIPF3* was highly expressed in immune organs and gave a response to immune stimulation. Further, ETS2, ZBTB4, and ZBTB14 all played important regulation roles for *pYIPF3* transcription. Combined with its localization of Golgi apparatus, it led us to a hypothesis about porcine *YIPF3* in the immune system: The high expression of *YIPF3* contributed to enhancing ER to Golgi transport of proteins, thus adapting to the immune stimulation. The function and regulation profiles about *pYIPF3* provides useful information in pig breeding, especially for disease resistance.

## Figures and Tables

**Table 1:** Primer pairs used for PCR amplification

Name	Sequence	Position1)	Size (bp)
Primers for promoter verification			
YIPF-proF	GAGACCTTCAGTTGCCTTCTTCCAG	−1,789	2,281
YIPF-proR	CTAAGTTGTGCGGAAAGCAAGAGC	+492	-
Primers for TSS			
YIPF-TSS(1)F	TCTCCTTTTTGCGTTCCG	+51	-
YIPF-TSS(2)F	GTCAGTTTTAACCAAACATCC	+25	-
YIPF-TSS(3)F	ATACCGGAAGTCAGTTTTAAC	+16	-
YIPF-TSS(4)F	TCCTCAGATACCGGAAGTC	+9	-
YIPF-TSS(5)F	CTCCGTCCACGTCCTCAGA	−3	-
YIPF-TSS(6)F	ATCTGCCCCGCCTCCGTC	−14	-
YIPF-TSS(7)F	AAATATCGCGAGAATCTGCC	−27	-
YIPF-TSSR	CATCGAATCCTCCCCATTC	+240	-
Primers for real time-PCR			
YIPF-RT-F	CTGCGTGAGGAAGAAGTGGAT	Extron1	176
YIPF-RT-R	AGGATGTCGATGTTGGCGTAC	Extron2	-
β-actin -F	GGATGCAGAAGGAGATCACG	-	-
β-actin -R	CTCGTCGTACTCCTGCTTGC	-	-
GAPDHF	CGTCCCTGAGACACGATGGT	-	-
GAPDHR	GCCTTGACTGTGCCGTGGAAT	-	-
Primers for subcellular location			
YIPF-L-F	CTCGAGACATGGCAACTCCG (2)	5′-UTR	-
YIPF-L-R	AAGCTTGTCAGTGTGACTGTAG	3′-UTR	-
Primers for promoter analysis			
YIPF-Prom(1)F	ACACTCGAGGAGACCTTCAGTTGC	−1,789	1,877
YIPF-Prom(2)F	CTCGAGATCTGATGCGGAAGATC	−1,654	1,742
YIPF-Prom(3)F	AATCTCGAGAAACAGCTGGAGGAAG	−1,469	1,557
YIPF-Prom(4)F	ATTCTCGAGCAACCTGAAACGAAT	−1,109	1,197
YIPF-Prom(5)F	AGCCTCGAGGCGGTATCAATTAAG	−739	827
YIPF-Prom(6)F	TACCTCGAGTCACTAGAGTCGTGT	−589	677
YIPF-Prom(7)F	CAGCTCGAGTCATAGCCCGAATAG	−359	447
YIPF-Prom(8)F	GTCCTCGAGACTTACATTGCGAAC	−198	286
YIPF-Prom(9)F	CAACTCGAGACCTCCCGGAATC	−42	130
YIPF-PromR	CAGAAGCTTGAAAGAAAGGTGGGA	+74	-
Primers for site-directed mutagenesis			
YIPF-MutEtsF	CGGCTCCGCATTTGCAGCACAGCCTGAGC	−161	-
YIPF-MutEtsR	GCTCAGGCTGTGCTGCAAATGCGGAGCCG	−133	-
YIPF-MutZ4F	CAGCCTGAGCCGCGTCGATCTTCAACTCCAC	−142	-
YIPF-MutZ4R	GTGGAGTTGAAGATCGACGCGGCTCAGGCTG	−112	-
YIPF-MutZ14F	GAGTACCAAGCCTGCTCGAGCGTTCTAGTC	−101	-
YIPF-MutZ14R	GACTAGAACGCTCGAGCAGGCTTGGTACTC	−72	-

PCR, polymerase chain reaction; TSS, transcription starts site.

1) This position is relative to the TSS.
